# Pott's puffy tumor: rare complication of sinusitis^☆^

**DOI:** 10.1016/j.bjorl.2016.08.005

**Published:** 2016-08-24

**Authors:** Liliana Costa, Laurentino Mendes Leal, Fernando Vales, Margarida Santos

**Affiliations:** Centro Hospitalar São João – EPE, Departamento de Otorrinolaringologia, Porto, Portugal

## Introduction

Pott's Puffy Tumor (PPT) was first described in 1760 by Sir Percival Pott.^1^, ^2^, ^3^ It is a subperiosteal abscess of the frontal bone with osteomyelitis, and it is an extracranial complication of an acute frontal sinusitis.^2^, ^4^ It is more common in adolescents, due to increased vascularization in dyploic circulation of the frontal sinus in this age, which allows a more rapid spread of infection.^1^

## Case report

**Figure 1:**
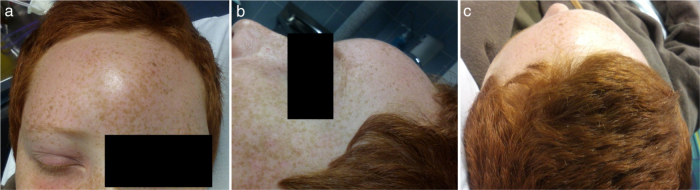
(a–c) Physical findings at admission in our hospital: right frontal swelling, with redness and fluctuation; and right periorbital celulitis.

**Figure 2:**
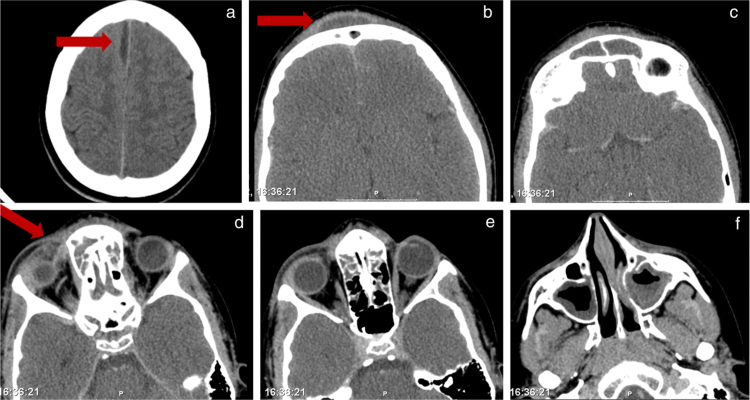
CT scan of brain, orbits and paranasal sinuses. (a) subdural and inter-hemyspheric empyema; (b) frontal subcutaneous abscess; (c) frontal sinusitis; (d–e) right orbital abscess and ethmoidal sinusitis; (f) maxillary sinusitis.

A 13-year-old male, with history of repeated superior airway infections, was admitted to another hospital with fever, headache and photophobia with 11 days of evolution, with a presumptive diagnosis of acute meningitis (lumbar puncture revealed 93% of polymorphonuclear cells in CSF). Empirical intravenous antibiotics were started: ceftriaxone and vancomycin. At 7th day, unfavorable evolution occurred: fever reappearance, strong headache, and right periorbital swelling and frontal right tumefaction. The patient was then transferred to our institution – a tertiary hospital. At admission, he only complained of pain in right eye. At physical examination, he was apyretic, but had noticeable edema, redness and cellulitis around the right eye. A frontal right tumefaction with fluctuation was palpable (Fig. 1). There was no rhinorrhea in anterior or posterior rhinoscopy. Meningeal signs were negative, as well as other focal neurological signs. Analytically, Hb = 11.7 g/dL, without leukocytosis, but increased C-reactive protein (79.9 mg/L). A Computed Tomography (CT) of paranasal sinuses, brain and orbits was performed and showed pansinusitis complicated with frontal subcutaneous abscess, inter-hemispheric empyema, and right orbital abscess (Fig. 2).

**Figure 3:**
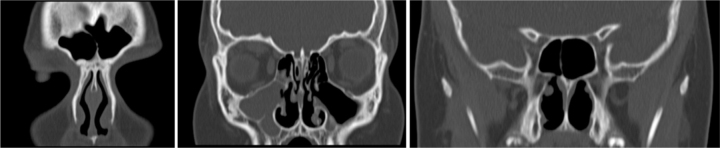
CT scan of paranasal sinuses (post-operative) showing only opacity of right maxillary sinus.

The patient was submitted to urgent multidisciplinary surgery: otolaryngologists drained frontal subcutaneous abscess with right supracilliary approach and frontal sinus trepanation; neurosurgeons performed a frontal craniotomy with drainage of interhemispheric and frontal empyema; and opfthalmologists drained orbital abscess through right orbitotomy. It was instituted empirical intravenous antibiotics: vancomycin, ceftriaxone and metronidazole, analgesia, nasal decongestant, and nasal washes. Although the patient showed good evolution after these surgeries, at 27th day after admission, it was performed a functional endoscopic sinuses surgery – FESS (right anthrostomy, anterior ethmoidectomy and frontal sinusotomy), bilateral inferior turbinoplasty and adenoidectomy. The post-operative period occurred without problems. The patient evolved favorably. Post-operative CT scan (2 months after FESS) only showed opacity of right maxillary sinus, in an asymptomatic patient (Fig. 3). There were no microbiological results of pus samples of surgeries. The patient was discharged at 43rd day, asymptomatic, after completing a 7-week course of ceftriaxone and vancomycin and a 6-week course of metronidazole.

## Discussion

Frontal acute sinusitis may have intra or extracranial complications through venous dissemination (most frequent) with thrombophlebitis of dyploic veins and septical embolus;^2^, ^4^ or by direct extension,^4^ which is possible in three directions: through posterior wall causing epidural empyema or abscess, subdural empyema, brain abscess or meningitis; through the anterior wall causing a PPT; and/or through the inferior wall with orbital complications.^1^, ^5^ It has been described that 85% of patients with PPT have also intracranial involvement.^6^ The presence of complications may be due to failure in early diagnosis of this entity,^7^ which is rare nowadays, since few cases have been described in the post-antibiotic age.^3^ Symptoms and signs usually present are headache, purulent rhinorrhea, fever, and frontal tumefaction with fluctuation, and sometimes periorbital cellulitis.^1^, ^8^ The gold standard exam is high-resolution CT that confirms the presence of osteomyelitis of external wall of frontal sinus that indicates PPT and reveals the presence of intracranial or intraorbital complications.^9^, ^10^ The most frequent bacterial agents implied are S*taphylococcus aureus*, *Streptococcus spp*, and anaerobes.^1^, ^2^, ^10^ Often the infection is polymicrobial. The cultures may be negative when previous antibiotics are instituted.^3^ Treatment is medical and surgical.^1^, ^2^, ^3^, ^4^, ^5^ Broad spectrum intravenous antibiotics with good penetration in CNS and anaerobic coverture must be instituted for at least 6–8 weeks (most commonly used are clyndamycin, ceftriaxone, metronidazole, vancomycin), and surgical drainage of affected areas must be done,^1^, ^5^ often requiring a multidisciplinary approach, including otorhinolaryngology, neurosurgery and ophthalmology.

## Conclusion

PPT is a rare entity due to a subperiosteal abscess often associated with a complication of frontal sinusitis. Undiagnosed or partially treated frontal sinusitis can lead to PPT. The case we presented is even more unusual because the tumor became complicated with orbital and intracranial involvement. Rapid medical and surgical interventions are required to prevent these complications that increase morbidity and mortality.

## Conflicts of interest

The authors declare no conflicts of interest.
